# Analysis of long non-coding RNAs associated with disulfidptosis for prognostic signature and immunotherapy response in uterine corpus endometrial carcinoma

**DOI:** 10.1038/s41598-023-49750-6

**Published:** 2023-12-14

**Authors:** Bohan Li, Xiaoling Li, Mudan Ma, Qing Wang, Jie Shi, Chao Wu

**Affiliations:** 1 Department of Gynecology and Oncology, Inner Mongolia Medical University, Affiliated Cancer Hospital, 42 Zhaowuda Road, Saihan District, Hohhot, 010000 Inner Mongolia China; 2 Department of General Surgery, Inner Mongolia Medical University, Affiliated Cancer Hospital, 42 Zhaowuda Road, Saihan District, Hohhot, 010000 Inner Mongolia China; 3 Department of Gynaecology and Obstetrics, Xi’an No. 3 Hospital, The Affiliated Hospital of Northwest University, No. 10, East Section of Fengcheng Third Road, Weiyang District, Xi’an, 710018 Shaanxi China; 4 Department of Pancreatic Cancer, Tianjin Medical University Cancer Institute and Hospital, National Clinical Research Center for Cancer, Key Laboratory of Cancer Prevention and Therapy, Tianjin’s Clinical Research Center for Cancer, Huanhu West Road, Hexi District, Tianjin, 300060 China

**Keywords:** Cancer, Gynaecological cancer

## Abstract

Disulfidptosis, the demise of cells caused by the abnormal breakdown of disulfide bonds and actin in the cytoprotein backbone, has attracted attention in studies concerning disulfide-related cell death and its potential implications in cancer treatment. This study utilized bioinformatics to detect disulfidptosis associated lncRNA prognostic markers (DALPMs) with Uterine Corpus Endometrial Carcinoma (UCEC)-related to investigate the correlation between these indicators and the tumor immune microenvironment. The RNA sequencing data and somatic mutation information of patients with UCEC were obtained from the Cancer Genome Atlas (TCGA) database. Patients were randomly divided into Train and Test groups. The findings revealed a potential prognostic model comprising 14 DALPMs. Both univariate and multivariate Cox analyses demonstrated that the model-derived risk score functioned as a standalone prognostic indicator for patients. Significant disparities in survival outcomes were observed between the high- and low-risk groups as defined by the model. Differences in tumor mutational burden (TMB), tumor immune dysfunction and exclusion (TIDE), and tumor microenvironment (TME) stromal cells between patients of the high- and low-risk groups were also observed. The forecast model comprising long non-coding RNAs (lncRNAs) associated with disulfidptosis can effectively anticipate patients' prognoses.

Uterine Corpus Endometrial Carcinoma (UCEC) is a prevalent gynecological cancer among females, presenting a significant risk to both physical and psychological well-being^1^. Based on the most recent cancer data in the United States, endometrial cancer is the fourth most common type of cancer in terms of new cases and ranks sixth in terms of total cancer-related fatalities. Approximately 66,200 individuals are diagnosed with endometrial cancer each year, resulting in around 13,030 deaths^2^. Furthermore, research has indicated that the chances of survival for females diagnosed with UCEC have been stagnant over the last four decades^3^. In contrast to other types of cancer, UCEC is frequently characterized by postmenopausal vaginal bleeding, which is a major symptom^4^. Furthermore, the occurrence of UCEC is steadily increasing, in part due to the growing occurrence of obesity^5^. Therefore, there is an urgent need to advance the diagnosis and treatment of endometrial cancer. Developing new diagnostic markers that can improve patient outcomes and guide treatment decisions is crucial in addressing this issue.

According to Ingenbleek and Kimura^6^, sulfur ranks as the seventh most plentiful component in the human body, mainly acquired through the consumption of food^7^. Sulfur is essential for living organisms and is involved in numerous metabolic and catalytic activities^8^. The tertiary and quaternary structures of sulfur-containing molecules are determined by disulfide bonds, which provide thermal stability and resistance to physicochemical deformation^9^. When cells are deprived of glucose, they undergo a unique form of cell death called disulfidptosis^10^, a process that involves the abnormal collapse of disulfide bonds and actin in cytoskeletal proteins, mediated by the uptake of cysteine through SLC7A11. Disulfidptosis is an unprecedented form of cellular demise with the potential to expedite the demise of cancerous cells by altering the structure of cytoskeletal proteins^11^. In addition, disulfidptosis potentially has ramifications in the realm of cancer treatment^12^. Nevertheless, further investigation is necessary to gain deeper insights into this mechanism; delving into long non-coding RNAs (lncRNAs) may offer a potential new treatment strategy.

Although a considerable amount of RNA is transcribed from the human genome, only a minor proportion is responsible for protein encoding^13^. The remaining transcripts are commonly known as non-coding RNAs (ncRNAs)^14^. These ncRNAs were first regarded as non-operational transcriptional ‘noises’^15^, but were found to have biological functions^16^. LncRNAs, exceeding 200 nucleotides in length^17^, have a significant impact on the regulation of chromatin dynamics, cellular proliferation, and organismal maturation^18^. Evidence suggests that modified lncRNA expression can initiate alterations in the growth and spread of tumor cells, indicating that lncRNA might have a role in either promoting or inhibiting cancer progression^19^. Meanwhile, research has found a close relationship between lncRNA and disulfidptosis. For example, lncRNA FLVCR1-AS1 mediates the miR-23a-5p/SLC7A11 axis to promote malignant behavior in cervical cancer cells; The downregulation of lncRNA SLC7A11-AS1 reduced the expression of NRF2/SLC7A11 and inhibited the progression of colorectal cancer cells. It should be pointed out that SLC7A11 is the core gene of disulfidptosis^20,21^. In addition, there is evidence to suggest an association between disulfidptosis gene related lncRNAs and cancer, which may guide the treatment and prognosis of cancer to some extent^22,23^. Hence, disulfidptosis related lncRNA is a potential indicator and treatment strategy for patients with cancer.

In this study, we developed a predictive model using lncRNAs associated with disulfidptosis to predict the prognosis of patients with UCEC and performed immune correlation analysis. The findings of our study introduce novel possibilities and concepts for UCEC research.

## Materials and methods

### Collection and collation of data

Data on RNA sequencing and somatic mutations of the individuals were obtained from the Cancer Genome Atlas (TCGA) (https://portal.gdc.cancer.gov) database. Patient clinical data were gathered from the TCGA and the University of California Santa Cruz Xena (UCSC Xena) (http://xena.ucsc.edu/) databases. After acquiring the clinical data, we compiled the information by removing samples that lacked survival information to guarantee the precision of subsequent analyses. We acquired annotation data for lncRNAs from the GENCODE website (https://www.gencodegenes.org/). Additionally, the literature provided us with the ten disulfidptosis-associated genes (GYS1, NDUFS1, OXSM, LRPPRC, NDUFA11, NUBPL, NCKAP1, RPN1, SLC3A2, and SLC7A11)^10^.

### Acquisition of disulfidptosis-associated lncRNA

The sequencing data for UCEC patients were obtained using the strawberry Perl (5.30.0.1). Subsequently, the expression data for both lncRNA and disulfide genes in UCEC patients were acquired. We utilized the R package “limma” to conduct correlation tests between the expression data of every lncRNA and the gene expression data of the patients related to disulfidptosis. The filter condition was set as corFilter > 0.3 and *p* < 0.001. To depict the co-expression correlation between genes related to disulfidptosis and lncRNAs associated with disulfidptosis, we utilized the R packages “dplyr,” “ggalluvial,” and “ggplot2” to generate Sankey plots.

### Establishing a prognostic model of disulfidptosis-associated lncRNA prognostic markers for UCEC

Patients were randomly divided into two groups, namely Train and Test, with equal proportions using the R package “caret.” Subsequently, we performed univariate Cox analysis to identify lncRNAs linked to disulfidptosis that exhibited a correlation with the prognosis of UCEC patients (*p* < 0.05). We utilized the R package “glmnet” to perform LASSO-Cox regression analysis, aiming to mitigate overfitting by setting the cross-validation penalty parameter λ to 10. Subsequently, multivariate Cox analysis was used to identify the most suitable disulfidptosis associated lncRNA prognostic markers (DALPMs) for modeling. To demonstrate the regulatory connection between DALPM and disulfidptosis genes, we generated heat maps utilizing the R packages “limma,” “reshape2,” “tidyverse,” and “ggplot2.” Finally, the model was constructed and the risk score for each patient was computed using DALPMs. The calculation of the risk score was performed in the following manner.

1 $$Risk\;score = \mathop \sum \limits_{i = 1}^{n} \left( {Coef\left( i \right) \times Expr\left( i \right)} \right)$$

In this context, Coef(i) represents the regression coefficient of lncRNA(i), while Expr(i) represents the standardized expression level of lncRNA(i). Based on the median risk score of patients in the Train group, the patients were categorized into groups of high risk and low risk. We utilized the R software package called “pheatmap” to examine the dispersion of survival status and its correlation with individual DALPMs as risk scores gradually escalated.

### Precision assessment of the model created using DALPMs

The model's verification involved a comprehensive assessment of the risk score determined by the model. Initially, we employed principal component analysis (PCA) with the assistance of the R package ‘scatterplot3d’ to visually represent the distinction between DALPMs and other variables in relation to patients categorized as high and low risks, then employed the R libraries ‘survival’ and “survminer” to determined possible variations in overall survival (OS) and progression-free survival (PFS) among individuals with varying risk scores. Furthermore, the risk score underwent independent prognostic analysis through univariate and multivariate Cox analyses using the R package “survival.” We generated Receiver Operating Characteristic (ROC) curves utilizing the R packages “survival,” “survminer,” and “timeROC” to assess the precision of our risk scores in predicting patient outcomes. For the same objective, the C-index analysis was conducted utilizing the R packages “dplyr,” “survival,” “rms,” and “pec.”

### Nomogram composition and accuracy detection

To create a nomogram that offers a thorough and precise prognosis for individuals diagnosed with UCEC, we utilized various R packages (“survival,” “regplot,” “rms,” and “survcomp”). This nomogram incorporated all relevant patient clinical factors to accurately predict individual patient survival. We utilized the calibration curve to assess the precision of our developed nomogram.

### Model prediction of the survival of patients at various clinical stages

To assess the accuracy of the model, we determined possible difference of survival status among different risk scores UCEC patients at different clinical stages by plotting survival curves with the help of the R packages “survival” and “survminer.”

### Analysis of GO, KEGG, and GSEA

Using the R package “limma,” we identified differentially expressed genes (DEGs) by comparing patients in the high and low risk groups. The criteria for identifying DEGs were a log2 |fold change|> 1 and a false discovery rate < 0.05. To better understand the biological role and pathway of these DEGs, we employed the R packages “clusterProfiler” and “org.” The packages used are “Hs. eg. Db,” “enrichplot.” We utilized software packages for the execution of Gene Ontology (GO), Kyoto Encyclopedia of Genes and Genomes (KEGG), and Gene Set Enrichment Analysis (GSEA). The gene lists 'c5. go. v7. 4. symbols. gmt' and 'c2. cp. kegg. v7. 4. symbols. gmt' were obtained from the Molecular Signature Database (MsigDB) (https://www.gsea-msigdb.org/gsea/msigdb/).

### Tumor microenvironment and immune invasion analysis

The stromal score, immune score, and ESTIMATE score of each patient were calculated using the ESTIMATE algorithm with R packages “limma” and “estimate.” We employed the R packages ‘reshape2’ and ‘ggpubr to examine the aforementioned scores among the high and low risk groups. Subsequently, we generated a violin plot to investigate potential disparities among the three patients in the high and low risk groups. Moreover, the examination of immune cell infiltration in each individual was conducted utilizing the R software package called “CIBERSORT,” and the outcomes were presented visually with the aid of the R packages “reshape2” and “ggpubr.” Furthermore, we conducted examination to assess immune-related functions and generated box plots to display the outcomes, utilizing the R packages “limma,” “GSVA,” and “GSEABase.”

### Somatic mutation data analysis

Using PERL, the somatic mutation data of patients were gathered, extracting the data for each patient and calculating the tumor mutational burden (TMB) value. To determine the genes with the highest number of mutations, we used the software package “maftools” to collate the somatic mutation data of patients at high and low risk for UCEC. Next, we presented the survival differences between high and low TMB patients, as well as the survival differences among patients when comprehensively evaluating their TMB and risk scores.

### Tumor immune evasion, immunotherapy response, and drug susceptibility analysis

The Tumor Immune Dysfunction and Exclusion (TIDE) scoring file of patients was obtained from the TIDE website, which focuses on TIDE and used to determine possible variation in the response to immune checkpoint blocking among patients in both groups, using the software package “ggpubr.”We performed a drug susceptibility analysis to assess the difference in drug susceptibility between patients in high and low risk groups. The evaluation criterion was the IC50 value, representing the semi-inhibitory concentration of the drug being tested. Lower IC50 measurement indicates better drug sensitivity. For this analysis, we employed the R package “pRRophetic.”^24,25^

### Statistical analysis

Statistical analyses were conducted utilizing R software (version 4.2.1), considering *p* < 0.05 as the threshold for statistical significance. Pearson's correlation test was used to investigate the connections between genes associated with disulfidptosis and lncRNAs related to disulfidptosis. The chi-square test was used to compare categorical data across different groups. To compare the distinction between the two groups, the Wilcoxon rank-sum test was employed. The log-rank test was employed to assess the disparity in survival using the Kaplan–Meier (KM) curve.

## Results

### Acquisition of disulfidptosis gene co-expression lncRNA

**Figure 1:**
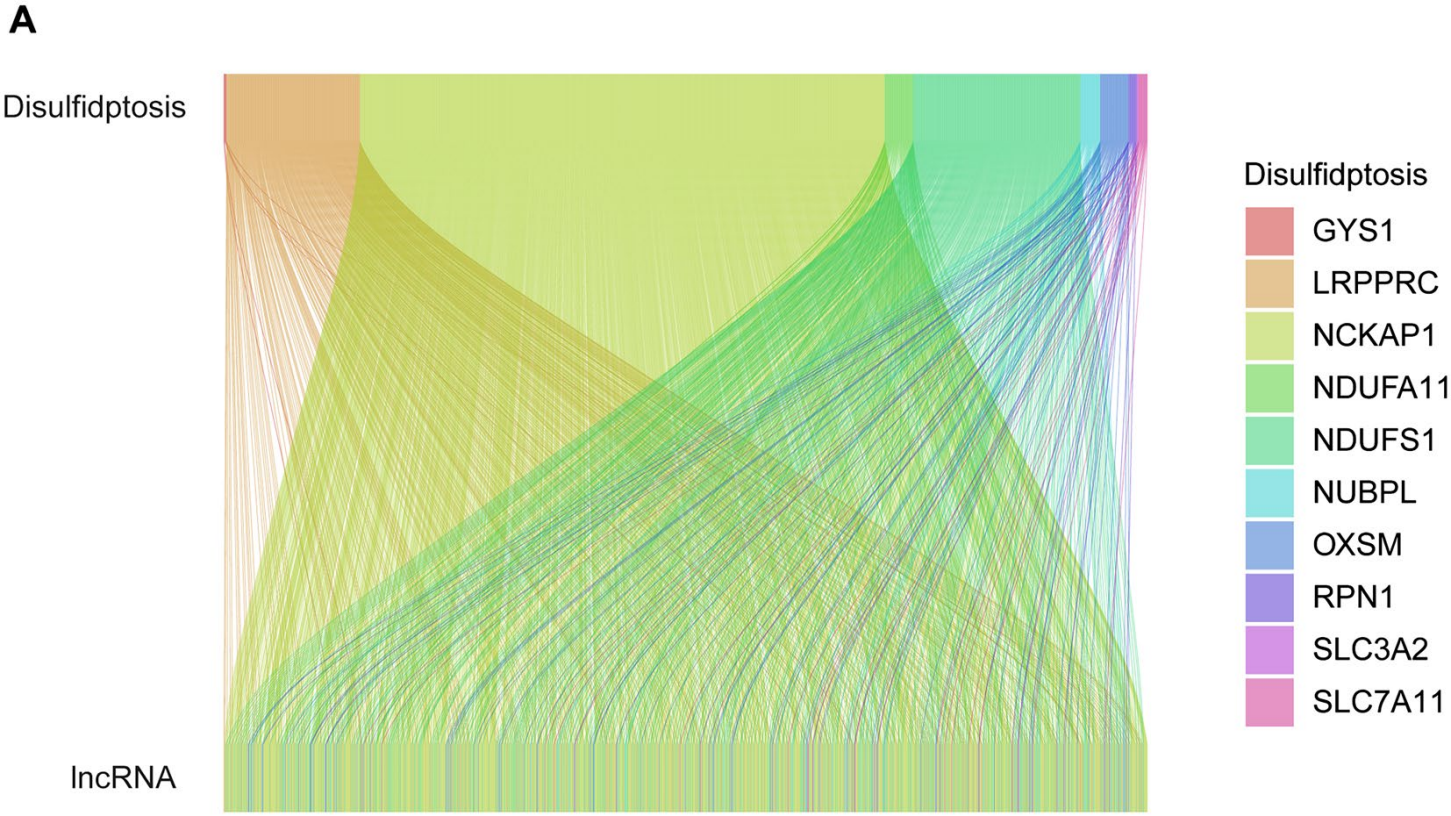
Acquisition of disulfidptosis gene co-expression lncRNA. (**A**) The Sankey plot showing 10 disulfidptosis core genes and their co-expressed lncRNAs.

A total of 16,877 lncRNAs were extracted from the TCGA-UCEC RNA-seq sequencing data. Co-expression of these 10 disulfidptosis genes with lncRNAs showed 1,136 lncRNAs associated with disulfidptosis death, with a significant correlation (|Pearson R|> 0.3 and *p* < 0.001). Our results are visualized using a Sankey plot, as shown in Fig. 1.

### Establishment of the DALPM model

A total of 543 patients were divided into two groups: Train (n = 272) and Test (n = 271). The validation results for clinical grouping indicated that our grouping was justified, and there were no disparities between the two groups in terms of diverse clinical factors (Table 1).

**Table 1:** Clinical grouping validation for the train and test groups.

Covariates	Type	Total	Test	Train	*p* Value
Age	≤ 65	306 (56.35%)	161 (59.41%)	145 (53.31%)	0.177
Age	> 65	235 (43.28%)	109 (40.22%)	126 (46.32%)	
Age	Unknown	2 (0.37%)	1 (0.37%)	1 (0.37%)	
Gender	FEMALE	543 (100%)	271 (100%)	272 (100%)	0.9658
Grade	G1	99 (18.23%)	58 (21.4%)	41 (15.07%)	0.1635
Grade	G2	121 (22.28%)	59 (21.77%)	62 (22.79%)	
Grade	G3	312 (57.46%)	149 (54.98%)	163 (59.93%)	
Grade	Unknown	11 (2.03%)	5 (1.85%)	6 (2.21%)	
Stage	Stage I	339 (62.43%)	177 (65.31%)	162 (59.56%)	0.5077
Stage	Stage II	52 (9.58%)	22 (8.12%)	30 (11.03%)	
Stage	Stage III	123 (22.65%)	58 (21.4%)	65 (23.9%)	
Stage	Stage IV	29 (5.34%)	14 (5.17%)	15 (5.51%)	

**Figure 2:**
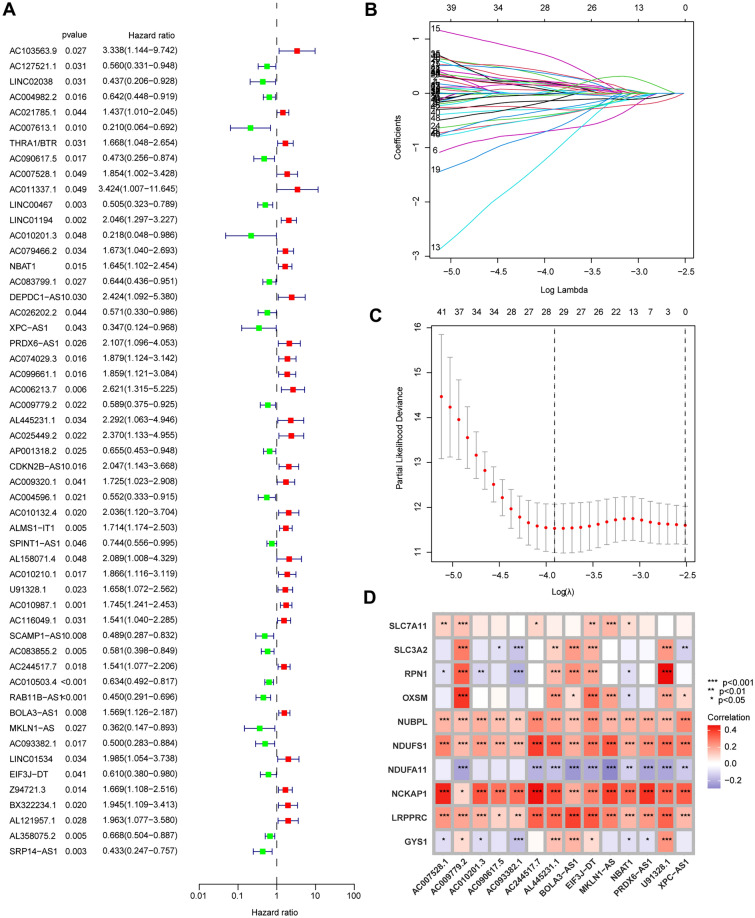
Establishment of the DALPM model. (**A**) A total of 53 disulfidptosis-associated lncRNAs independently associated with the prognosis of patients with UCEC were identified via univariate Cox screening. (**B**, **C**) LASSO regression analysis was used to screen for lncRNAs suitable for model construction. (**D**) Regulatory relationship between DALPMs and disulfidptosis core genes.

**Figure 3:**
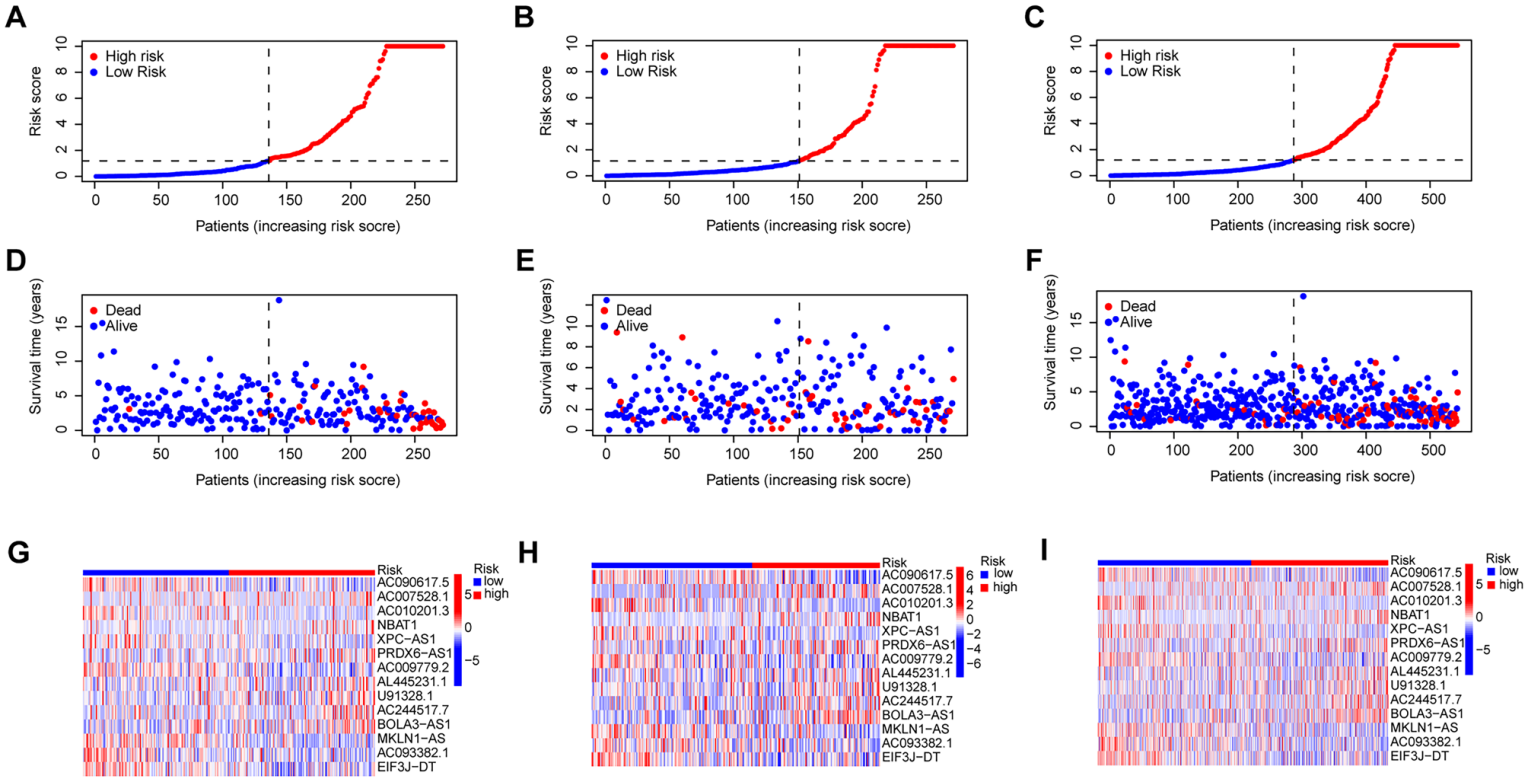
Distribution of survival status and DALPMs in patients with elevated risk scores. (**A**–**C**) Patient risk score distribution. (**D**–**F**) Distribution of patient survival status with risk score. (**G**–**I**) Distribution of DALPMs with risk scores.

The Train group was subsequently utilized for constructing the model, while the Test group and all patients were employed for testing. Initially, we conducted univariate Cox analysis on 1,136 lncRNAs associated with disulfidptosis identified through co-expression analysis. A total of 53 prognostically relevant genes were identified (Fig. 2A). Using LASSO regression analysis, we identified 29 lncRNAs that exhibited the most significant prognostic predictive values (Fig. 2B,C). Multivariate Cox analysis screened 14 DALPMs (AC090617.5, AC007528.1, AC010201.3, NBAT1, XPC-AS1, PRDX6-AS1, AC009779.2, AL445231.1, U91328.1, AC244517.7, BOLA3-AS1, MKLN1-AS, AC093382.1, and EIF3J-DT) and constructed a prognostic model. In addition, we created heat maps to illustrate the regulatory connection between DALPMs and disulfidptosis genes (Fig. 2D). We computed the risk score using the multivariate Cox regression formula: Risk score = (AC090617.5 × –1.0913) + (AC007528. 1 × 1.1325) + (AC010201.3 × –3.4564) + (NBAT1 × 1.4757) + (XPC-AS1 × -1. 2011) + (PRDX6-AS1 × 1. 0761) + (AC009779.2 × –0.8874) + (AL445231. 1 × 0.8907) + (U91328. 1 × 0.7976) + (AC244517. 7 × 0.7865) + (BOLA3-AS1 × 0.65885) + (MKLN1-AS × –1.1178) + (AC093382.1 × –1.1315) + (EIF3J-DT × –1.2246). Based on the median risk score of patients in the Train group, the patients were categorized into groups of high and low risks. As the risk score increased, the number of patients in the survival state gradually decreased, as shown in Fig. 3A–F. The expression of DALPMs in patients is depicted in Fig. 3G–I, which illustrates the relationship between risk score and DALPM expression. For example, the AC090617.5 gene demonstrates an inverse relationship with the risk score, whereas the BOLA3 − AS1 gene displays a direct correlation.

### Validation of model accuracy

**Figure 4:**
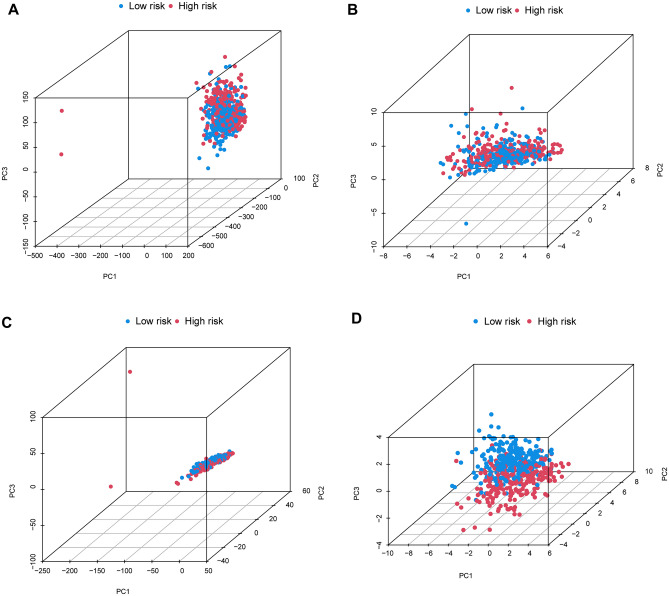
PCA analysis of all patients. (**D**) DALPMs had better discrimination in terms of patient risk compared with (**A**) all genes, (**B**) disulfidptosis genes, and (**C**) all disulfidptosis lncRNAs.

**Figure 5:**
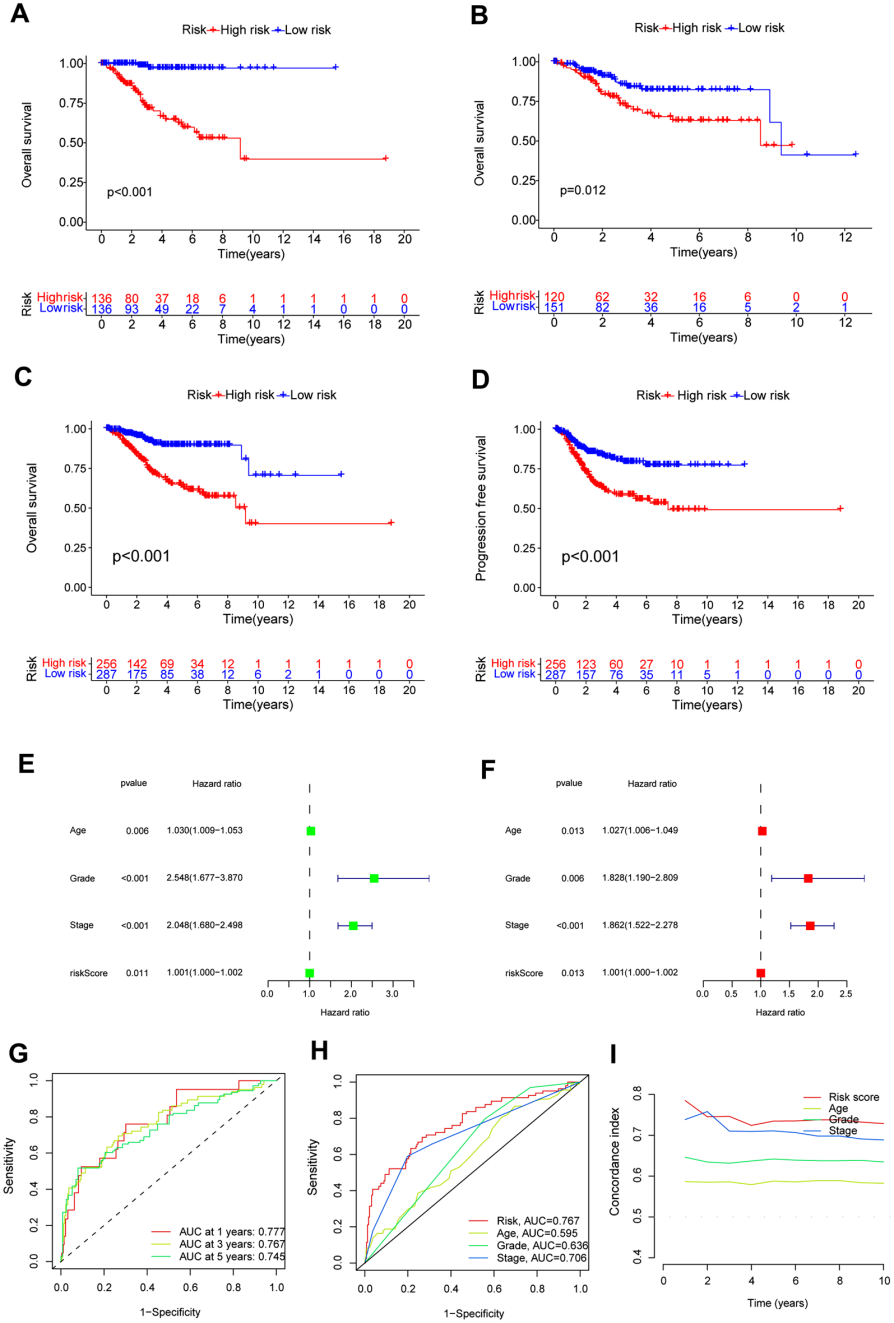
Validation of the accuracy of the model from multiple perspectives. (**A**–**C**) KM curves of OS in the Train, Test, and All groups. (**D**) KM curves of PFS in the All group. (**E**, **F**) Univariate and multivariate Cox analysis suggesting that risk score is an independent prognostic factor for UCEC. (**G**) DALPMs predicts 1-, 3-, and 5-year survival rates in patients with UCEC. (**H**) The ROC curve showing that risk scores have better predictive power compared with other clinical factors. (**I**) The C-index showing that risk scores have better predictive power compared with other clinical factors.

We examined the accuracy of the prognostic model created by DALPMs. PCA analysis (Fig. 4A–D) indicated that DALPMs demonstrated superior ability in identifying risk status when compared with all genes, disulfidptosis genes, and all disulfidptosis lncRNAs. According to the KM survival curve, patients in the high-risk group had lower OS and PFS rates compared with those in the low-risk group, suggesting a poorer prognosis for the former (Fig. 5A–D). Furthermore, the outcomes of univariate and multivariate Cox analysis indicated that the risk scores remained unaffected by UCEC (Fig. 5E, F). Based on the AUC of the ROC curve, the prognostic prediction of patients with risk scores yielded AUC values of 0.777, 0.767, and 0.745 for 1, 3, and 5 years, respectively (Fig. 5G). In comparison with other clinical factors in UCEC, the risk score demonstrated the highest AUC, suggesting that it had a notable advantage in prognostic prediction for patients with UCEC (Fig. 5H). These findings were supported by the C-index curve (Fig. 5I).

### Construction of the nomogram

**Figure 6:**
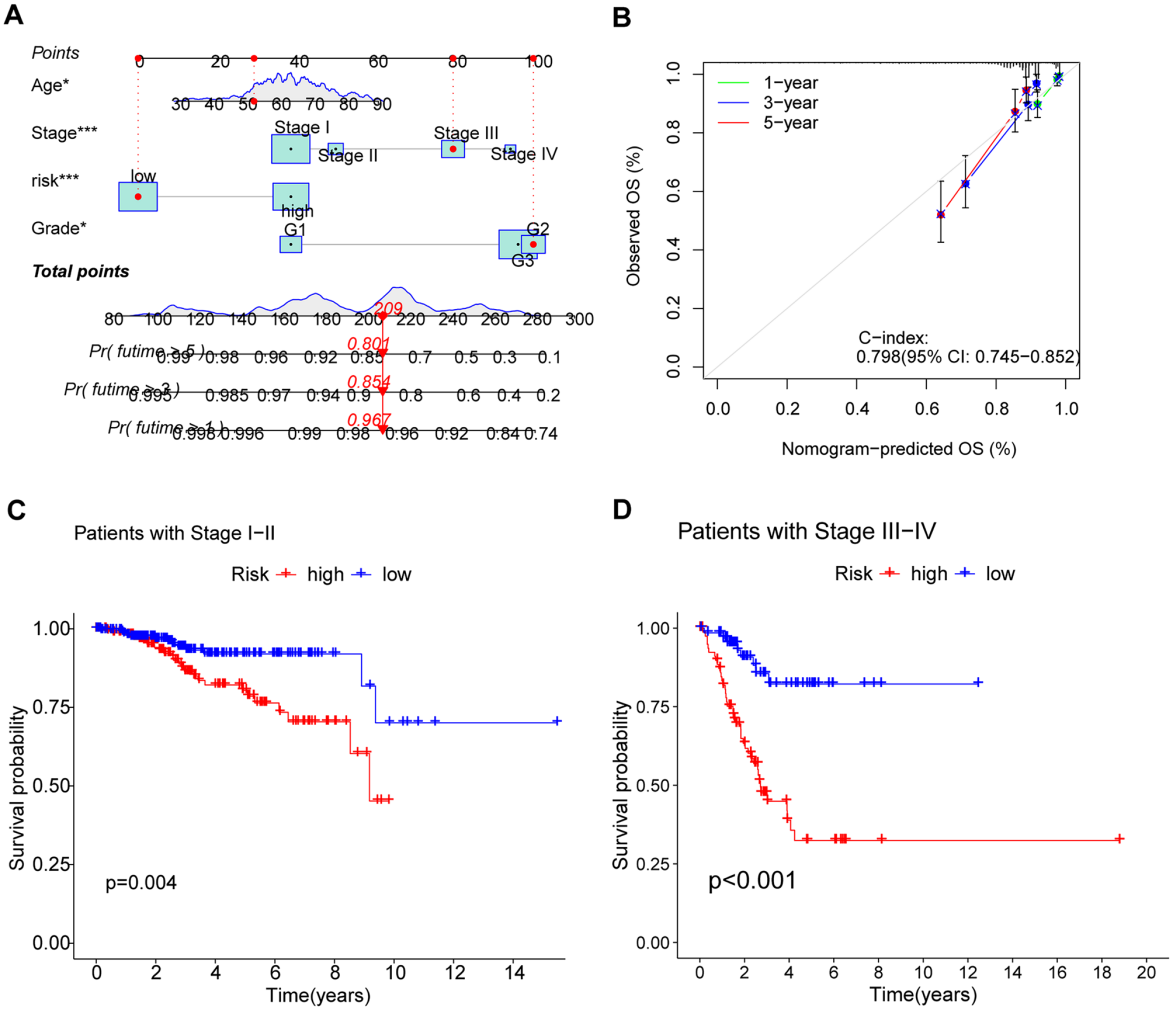
Construction of the nomogram and exploration of the correlation between risk scores and the clinical stage of UCEC. (**A**) The nomogram showing that clinical factors, including risk scores, can be scored to predict the survival of patients at 1, 3, and 5 years. (**B**) The calibration curve showing sufficient consistency between the actual results and the predicted results. (**C**, **D**) differences in survival between high and low risk groups of patients at different stages.

A comprehensive score was calculated for every patient, considering factors such as age, grade, stage, and risk score. Next, we created a corresponding nomogram to precisely forecast the 1-, 3-, and 5-year outlook for every individual (Fig. 6A). The accuracy of the prediction was further confirmed by the calibration curve, as shown in Fig. 6B.

### The correlation between risk scores and the clinical stage of UCEC

To further investigate the practicality of DALPMs, we examined their use in forecasting risks in various clinical phases. The results from the KM curve showed a notable variation in survival rates among patients with varying risk scores in clinical stages I-II and III-IV (Fig. 6C,D).

### Results from the analysis of GO, KEGG, and GSEA

**Figure 7:**
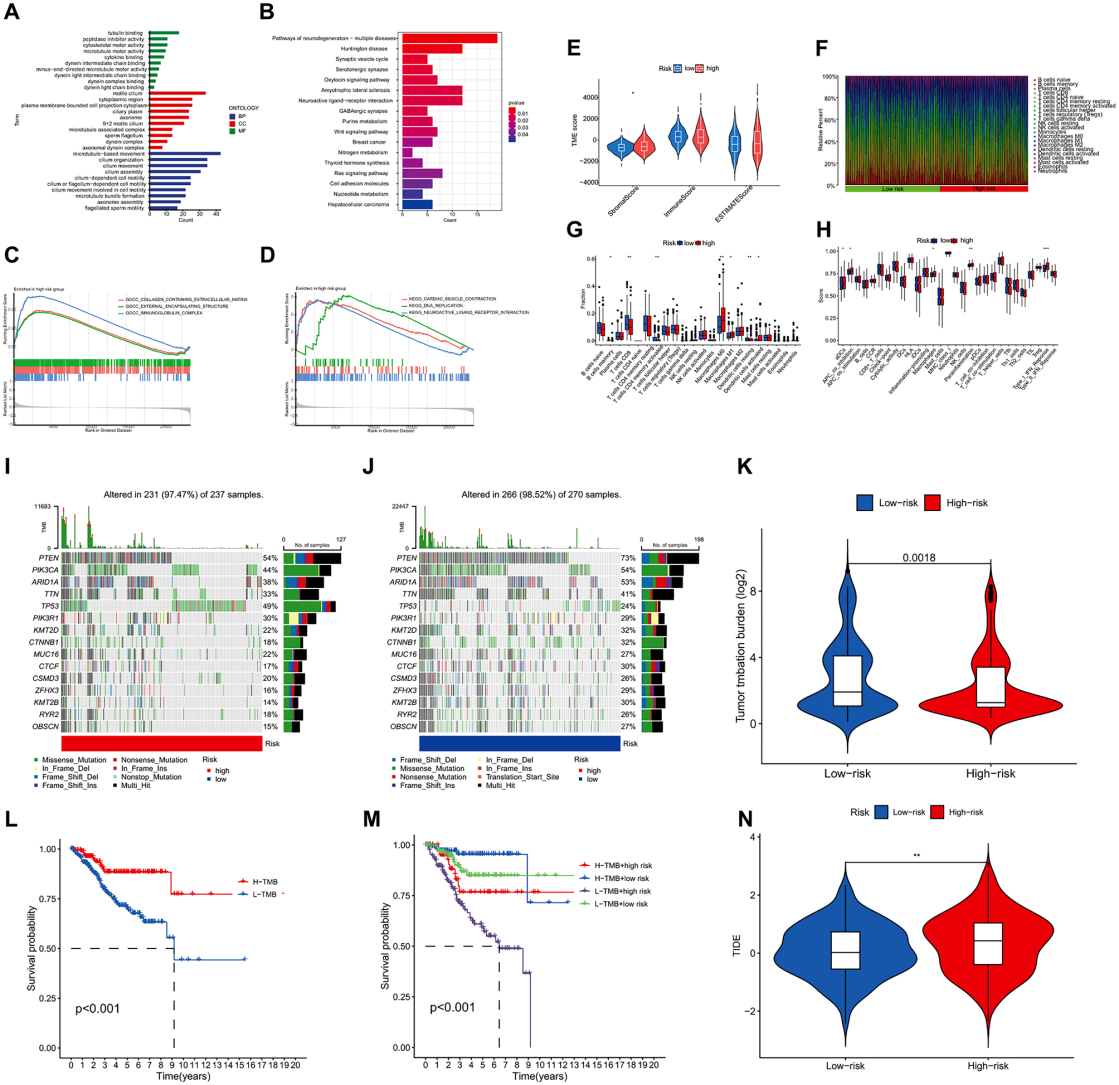
The results of GO, KEGG, GSEA, TME, immune invasion TMB, and TIDE analyses. (**A**) GO enrichment analysis revealed the top 10 biological functions in BPs, CCs, and MFs. (**B**) KEGG analysis revealed significantly enriched pathways. (**C**, **D**) GSEA analysis revealed the biological functions and pathways of the high risk group located in the top three. (**E**) Differences in stromal cells in patients in high and low risk groups. (**F**) Abundance of immune cell infiltration in all patients. (**G**, **H**) Differences in immune cells (B cells memory, T cells CD8, T cells CD4 memory activated, macrophages M0, macrophages M1, dendritic cells resting, and dendritic cells activated) and immune-related pathways (aDCs, APC co-inhibition, macrophages, parainflammation, and Type I IFN Response) in patients in high and low risk groups. (**I**, **J**) Somatic mutation in the high and low risk groups. (**K**) Differences in TMB between the high and low risk groups. (**L**) A significant difference in survival between the high and low risk groups of patients with TMB was observed. (**M**) Patients with low TMB and high risk have the worst survival. (**N**) Differences in TIDE between patients in the high and low risk groups were observed. *p < 0.05, **p < 0.01, ***p < 0.001.

A total of 512 DEGs were extracted from patients in the high- and low-risk groups. The findings from the analysis of GO enrichment indicated that the primary biological processes (BPs) enriched by DEGs included microtubule-dependent motion, organization of cilia, and ciliary motion. The primary cellular components (CCs) were motile cilia, cytoplasmic area, and cytoplasmic extensions bounded by the plasma membrane. Additionally, the main molecular functions (MFs) identified were binding to tubulin, as peptidase inhibitors, and functioning as a cytoskeletal motor (Fig. 7A). According to the KEGG enrichment analysis, the primary pathways enriched for DEGs were identified as pathways of neurodegeneration − multiple diseases, Huntington's disease, and synaptic vesicle cycle (Fig. 7B). In Fig. 7C, the collagen-containing extracellular matrix, external encapsulating structure, and immunoglobulin complex were the three highest-ranked CCs within the high-risk group. Figure 7D displays the findings of GSEA analysis; the high-risk group exhibited cardiac muscle contraction, DNA replication, and neuroactive_ligand receptor interaction as the top three pathways.

### Tumor microenvironment and immune invasion analysis

To examine variations in tumor microenvironment (TME) between the high- and low-risk groups, we computed the TME score for every individual. Figure 7E illustrates variations in stromal cells between the high- and low-risk groups, while Fig. 7F illustrates the infiltration of immune cells in all individuals. Furthermore, we examined the disparities in immune cell infiltration among patients classified as high risk and low risk. Figure 7G displayed variations in B cells memory, T cells CD8, T cells CD4 memory activated, macrophages M0, macrophages M1, dendritic cells resting, and dendritic cells activated, as indicated by the findings. Ultimately, we contrasted the variances in immune pathways among patients in the high- and low-risk groups. Significant differences in immune pathways, such as aDCs, APC co-inhibition, macrophages, parainflammation, and Type I IFN Response (Fig. 7H), were observed between the two groups.

### TMB analysis

Using somatic mutation data, the connection between TMB and the risk score was established. According to the waterfall chart, the low-risk group exhibited a higher gene mutation rate compared with the high risk group. Moreover, the PTEN, PIK3CA, ARID1A, TTN, TP53, PIK3R1, KMT2D, CTNNB1, MUC16, and CTCF genes were identified as the top 10 genes with the greatest likelihood of mutation in both high- and low-risk groups. Notably, PTEN, PIK3CA, and ARID1A exhibited the highest mutation probabilities, as shown in Fig. 7I,J. According to the findings from the violin plot, a notable disparity in TMB was observed between patients categorized in the high- and low-risk groups (Fig. 7K). In addition, we found that there were differences in survival between patients with high and low TMB (Fig. 7L), and the use of TMB and risk score in predicting patient prognosis had a more detailed and specific predictive effect (Fig. 7M), which may be a supplement to using TMB alone to predict patient prognosis.

### TIDE analysis

From the TIDE website, we obtained the UCEC patient's TIDE scoring file. Upon analysis, we observed variations in TIDE scores between the high- and low-risk groups (Fig. 7N). The group at greater risk displayed elevated TIDE scores, indicating that tumor cells in this category had a higher chance of evading immune monitoring, which could result in a less favorable reaction to immunotherapy.

### Drug susceptibility analysis

**Figure 8:**
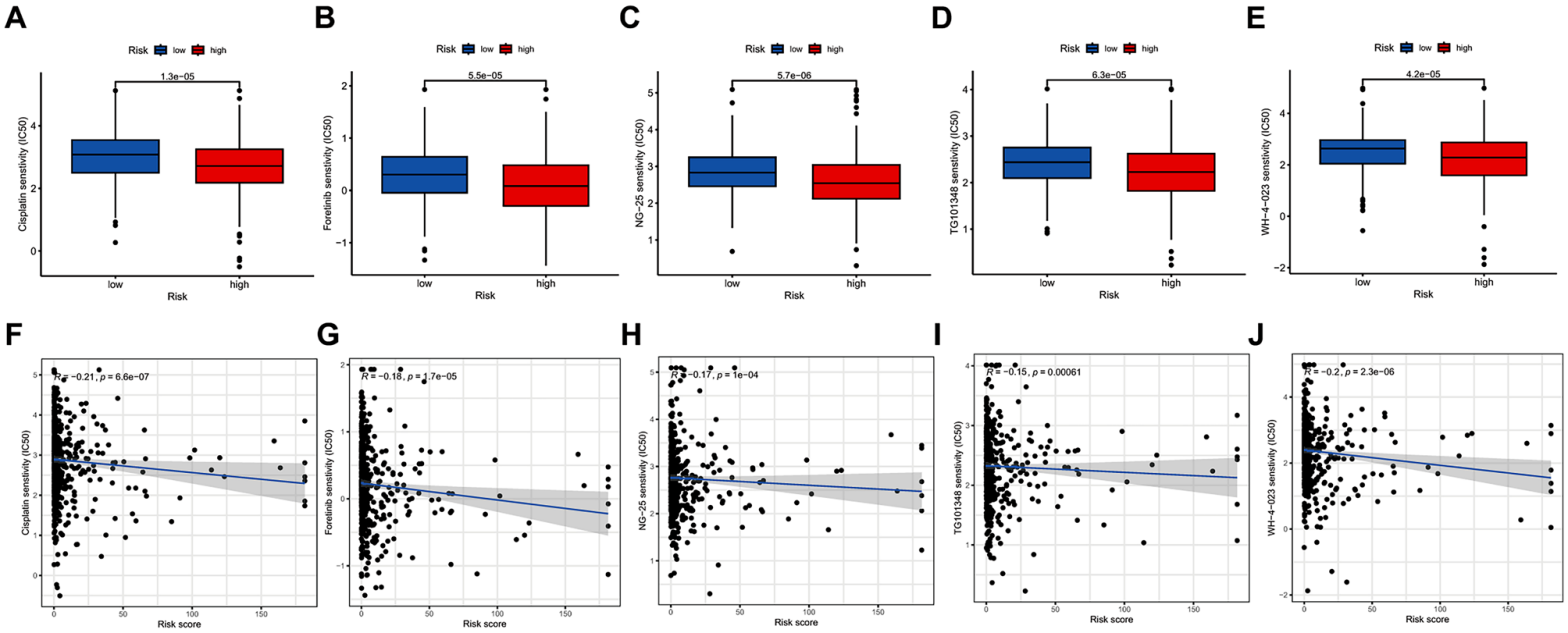
Drug susceptibility analysis. (**A**–**E**) Five drugs demonstrated lower IC50 values for patients in the high-risk group. (**F**–**J**) Correlation between the IC50 values and risk scores of the five drugs.

A total of 19 drugs with unique IC50 values were identified by comparing the IC50 values of various medications used to treat UCEC in high- and low-risk patients. Cisplatin, Foretinib, NG-25, TG101348, and WH-4-023 exhibited lower IC50 values and displayed a negative correlation with risk scores when administered to patients in the high-risk category (Fig. 8A−J). When treating patients in the low-risk group, several compounds including 5-Fluorouracil, AKT inhibitor VIII, AUY92, BAY 61–3606, CCT018159, CEP-701, EHT 1864, GSK1904529A, Mitomycin C, OSI-930, PHA-665752, Phenformin, Tipifarnib, and ZM-447439 exhibited lower IC50 values and showed a positive correlation with risk scores (Supplementary Figs. S1 and S2). These drugs warrant attention to ensure standardized treatment practices.

## Discussion

The prevalence of endometrial cancer is on the rise, and although surgery usually leads to positive outcomes for early-stage cases^26^, accurately assessing the likelihood of recurrence remains challenging^27^. Potential solutions are provided by prognostic markers, and recent lncRNA research demonstrates promise in addressing this challenge. For example, Sun et al. identified lncRNA prognostic indicators associated with copper-induced mortality in head and neck squamous cell carcinoma and developed a prognostic model for predicting patient outcomes^28^. The core gene of disulfidptosis is closely related to the regulation of lncRNA. In addition, cisplatin and paclitaxel hold potential for cancer treatment, including endometrial cancer. Studies have shown that the anti-tumor effects of these drugs may be exerted by reacting with intracellular disulfides^29,30^. The implication is the possibility of identifying lncRNA prognostic indicators associated with disulfide-induced mortality, which can be used to inform treatment choices for individuals diagnosed with endometrial cancer.

Using 14 DALPMs, we constructed a prognostic model to forecast the patients' prognosis in this research. By conducting co-expression analysis, a set of 1,136 lncRNAs linked to 10 disulfide death genes were extracted to acquire DALPM suitable for prognostic modeling. We obtained the 14 DALPMs for building our prognostic risk model through LASSO, univariate Cox, and multivariate Cox analyses. After examining these 14 DALPMs, we identified U91328.1, AC244517.7, AC009779.2, AC090617.5, AC093382.1, and BOLA3-AS1 as potential prognostic indicators for specific types of cancer^31–36^. Luo et al.^37^ reported a strong correlation between EIF3J-DT and the development of drug resistance in gastric cancer cells, while Chen et al.^38^ suggested MKLN1-AS as a therapeutic target for hepatocellular carcinoma (HCC). Pan et al.^39^ suggested that MKLN1-AS is highly probable to target miR-22-3p and induce carcinogenic effects, while Xue et al.^40^ and Wei et al.^41^ reported that NBAT1 has the potential to hinder the advancement of tumors like renal cell carcinoma and hepatocellular carcinoma. Our research revealed the presence of five new lncRNAs (AC007528.1, AC010201.3, XPC-AS1, PRDX6-AS1, and AL445231.1) that possess prognostic importance, emphasizing the originality and importance of our study. Additional research is necessary to explore the function and mechanisms of these lncRNAs in endometrial cancer.

To assess the necessity and accuracy of our model, we categorized patients into high and low risk groups by calculating the risk score using DALPMs. The KM curves for OS and PFS demonstrated a clear distinction in survival rates between patients, which implies that it is important to consider the treatment and prognosis of patients classified as high risk. We also conducted univariate and multivariate Cox analyses, revealing that the risk score independently predicts the prognosis of UCEC and generated ROC curves to compare the risk scores with other clinical factors currently in use. According to the ROC curves, the risk score exhibited the highest AUC value, indicating that utilizing the risk score as a predictor may provide a relatively more precise assessment of the prognosis for patients with UCEC. By combining risk scores with other clinical factors, the nomogram and calibration curve were created to estimate the 1-, 3-, and 5-year survival rates for patients. Furthermore, the risk scores can be used to differentiate the survival outcomes of patients with varying clinical stages. The precision examinations for our model produced extremely pleasing outcomes, suggesting its capability to precisely forecast the prognosis of patients diagnosed with UCEC.

The concept of TME, the existence of benign cells and constituents within tumor cells^42^, has been acknowledged for its association with inflammation and cancer^43^. With deepening research on TME, its importance has gradually been accepted. Cancer progression and treatment are closely linked to the tumor microenvironment^44,45^. For this purpose, we employed the ESTIMATE algorithm to compute the TME of the individuals, subsequently examining potential disparities between the three patients categorized into the high and low risk groups. The findings indicated variations in stromal cell assessment between the groups. Solid tumor progression is significantly influenced by stromal cells, especially cancer-associated fibroblasts, which also contribute to treatment response, angiogenesis, immune evasion, and drug resistance^46,47^. Our research indicates that there are differences in stromal scores between high and low-risk groups, and the stromal cell content seems to be higher in patients in the high-risk group. Therefore, this needs further attention from us.

Despite the absence of a general disparity in immune cell scores, a closer examination revealed discrepancies in particular immune cell populations, including memory B cells and activated CD4 memory T cells, among the high and low risk groups. The immune cells that are expressed differently in the TME could serve as potential targets for therapeutic interventions in UCEC. Immune cells with the ability to adapt, like B cells and T cells, have a significant impact on the TME^48–50^. Thorough investigation into these cells may aid in forecasting patient results and evaluating potential biological indicators. Helmink et al.^51^ found a significant increase in the presence of memory B cells within melanoma tumors in patients who positively responded to immune interventions. These findings have potential implications for the development of biomarkers and the identification of therapeutic targets. Similarly, Li et al.^52^ discovered a correlation between CENPF and markers of CD4 + memory T cells in melanoma. Increased expression of CENPF results in early exhaustion and immune suppression of CD4 + T cells, suggesting its potential as a predictive indicator for melanoma spread and a target for treatment. Therefore, exploring the immune cells expressed differently in TME of patients in high- and low-risk groups can reveal possible treatment targets for UCEC.

We examined the immune pathways that exhibited variations among patients in the two groups and observed variations in aDCs, APC-co-inhibition, macrophages, parainflammation, and Type-I-IFN-Reponse. Type-I-IFN encompasses a broad class of inflammatory cytokines released when the immune system is weakened^53^. Snell et al.^54^ reported that Type-I-IFN had both positive and negative effects. Type-I-IFN is closely related to radiotherapy and chemotherapy for cancer^55,56^. Nevertheless, the continuous Type-I-IFN-Reponse could potentially serve as a fundamental catalyst for immune dysfunction and the development of treatment resistance^57–59^. Our findings provide novel evidence suggesting the important role of Type-I-IFN Response in endometrial cancer based on our results.

Tumor mutational burden reflects the genetic mutation in patients. The examination of tumor mutation adherence indicated that the low-risk group exhibited a greater TMB compared with the high risk group, signifying a disparity in TMB between the two groups. PTEN, which plays a significant role in the advancement and therapy of cancer, exhibited the highest mutation rate among both groups. The inhibition of PTEN can enhance the release of exosomes and the spread of cholangiocarcinoma by impeding TFEB-mediated formation of lysosomes^60^. Shi et al.^61^ found that cancer-associated fibroblast-derived exosomal microRNA-20a inhibits the PTEN/PI3K-AKT pathway, thereby promoting cancer progression and chemotherapy resistance in non-small cell lung cancer. Endometrial cancer is significantly influenced by PTEN, and extensive molecular studies have consistently identified PTEN inactivation as the primary cause of endometrioid carcinoma, which is particularly true for individuals with PTEN-related disorders. After examining the prognosis of patients with TMB and risk scores as shared markers, we discovered that individuals with low TMB and elevated risk scores demonstrated the most unfavorable survival results.

The analysis of TIDE, which investigates the possibility of tumor evasion during immunotherapy, uncovered variations in TIDE scores among the groups at high and low risk. The group at increased risk demonstrated elevated TIDE scores, suggesting a higher probability of immune evasion during immunotherapy. This aligns with our earlier TME analysis. In addition, we performed drug susceptibility analysis to compare the effectiveness of medications among patients categorized as high and low risk groups. The findings from the drug sensitivity analysis revealed variances in the IC50 values of a total of 19 drugs among patients in both groups. Better treatment effects were observed in patients in the high-risk group when using drugs with lower IC50 values, such as Cisplatin, Foretinib, NG-25, TG101348, and WH-4-023, among the screened medications; therefore, these drugs deserve further attention. Our results indicate that our model can predict the prognosis and response to immunotherapy in patients with UCEC, potentially assisting in clinical decision-making.

In conclusion, disulfidptosis is a recently discovered form of cellular demise that is linked to cancer. lncRNAs affect cellular biological processes, consequently influencing the treatment of cancer. At present, there is still a lack of research on the disulfidptosis related lncRNAs in UCEC. By utilizing experimental and validation cohorts, our bioinformatics research allows for the investigation of prognostic biomarkers associated with disulfidptosis in UCEC while ensuring the model's reliability. We believe that our research provides a new perspective for the study of UCEC, and we believe that as the research continues to deepen, the prognostic markers we have identified will provide some guidance for clinical practice. We also believe that with the improvement of relevant experiments, this model has sufficient potential to be transformed into clinical tools to serve more patients. For example, we can quantify the expression of LncRNA within the model in the patient's body to comprehensively assess the patient's risk, estimate the patient's prognosis, and provide targeted guidance for treatment. However, our research may have the following limitations to some extent. We searched for UCEC-related datasets in the GEO database and found no dataset that met our analysis criteria. Therefore, we randomly organized the datasets from the TCGA repository to validate the precision of our findings. Furthermore, additional preclinical investigations are required to enhance the model's reliability prior to its implementation in clinical settings.

## Conclusion

In this study, we discovered 14 different DALPMs and utilized them to create a predictive model. The prognostic predictions for patients with UCEC can be accurate using this model, which has significant connections with tumor immunity and can partially guide UCEC treatment decisions. Our results provide a novel approach for future related studies.

### Supplementary Information

Supplementary Information.

## Data Availability

The datasets presented in this study can be found in online repositories. The names of the repository/repositories and accession number(s) can be found in the article/Supplementary Material.

## Abbreviations

DALPMs: Disulfidptosis associated lncRNA prognostic markers
UCEC: Uterine Corpus Endometrial Carcinoma
TCGA: The Cancer Genome Atlas
TMB: Tumor mutational burden
TIDE: Tumor immune dysfunction and exclusion
TME: Tumor microenvironment
lncRNAs: Long non-coding RNAs
UCSC Xena: University of California Santa Cruz Xena
PCA: Principal component analysis
OS: Overall survival
PFS: Progression-free survival
ROC: Receiver Operating Characteristic
DEGs: Differentially expressed genes
GO: Gene Ontology
KEGG: Kyoto Encyclopedia of Genes and Genomes
GSEA: Gene Set Enrichment Analysis
MsigDB: Molecular Signature Database
KM: Kaplan–Meier
BPs: Biological processes
CCs: Cellular components
MFs: Molecular functions
HCC: Hepatocellular carcinoma
