# Supplementary material for: Analysis of long non-coding RNAs associated with disulfidptosis for prognostic signature and immunotherapy response in uterine corpus endometrial carcinoma
Source: Sci Rep. 2023 Dec 14;13:22220. doi: 10.1038/s41598-023-49750-6 (PMC10721879; doi:10.1038/s41598-023-49750-6)
Supplement: Supplementary file 1 — Supplementary Information. [file 41598_2023_49750_MOESM1_ESM.pdf]

Supplementary Information

Analysis of long non-coding RNAs associated with disulfidptosis for prognostic signature and immunotherapy response in uterine corpus endometrial carcinoma

Bohan Li[1, 5], Xiaoling Li[2, 5], Mudan Ma[1, 5], Qing Wang[3, 5], Jie Shi[1*], Chao Wu[4*]

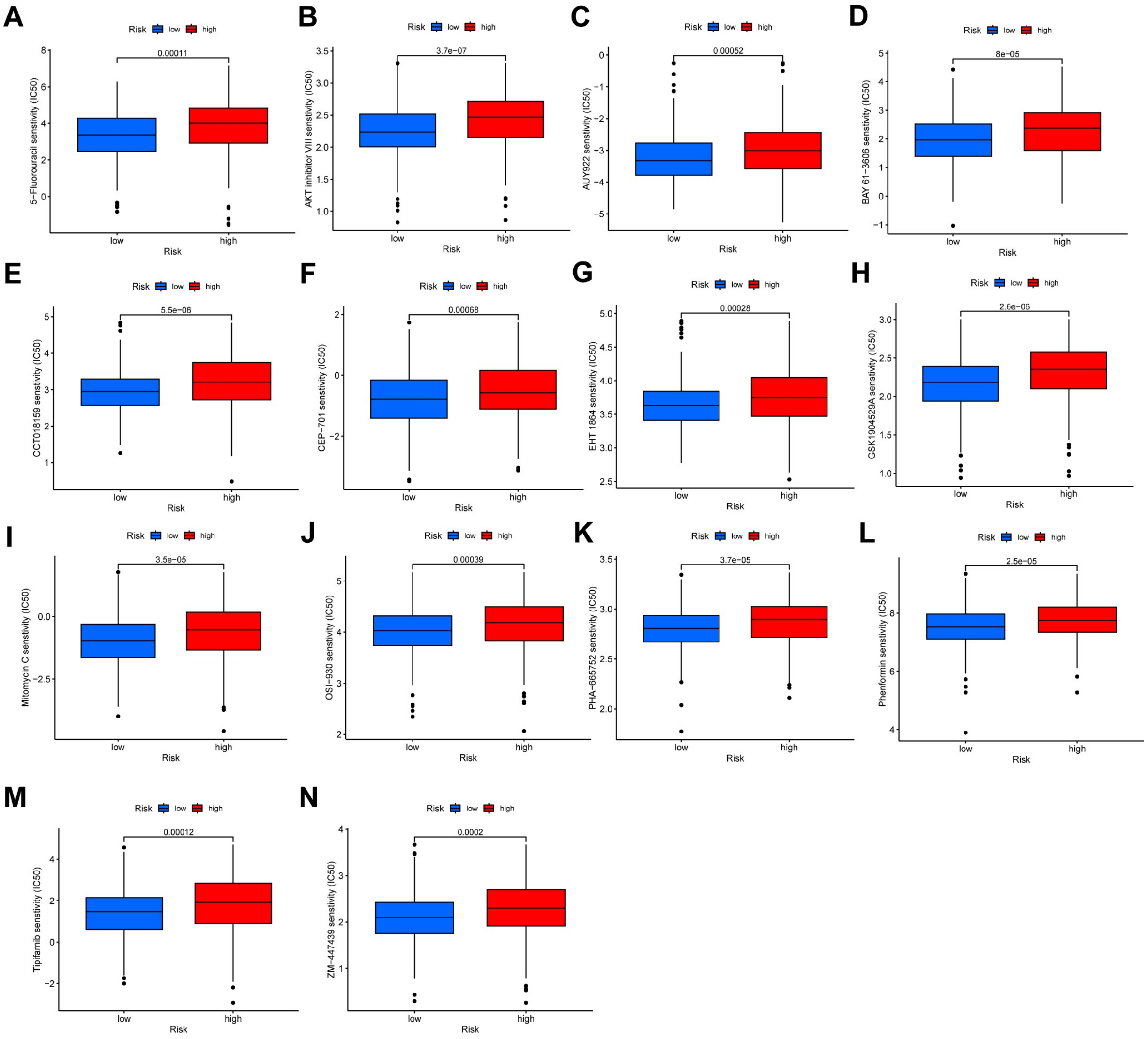

Supplementary Fig. S1 Drug susceptibility analysis. (A–N) Fourteen drugs demonstrated lower IC50 values for patients in the low-risk group

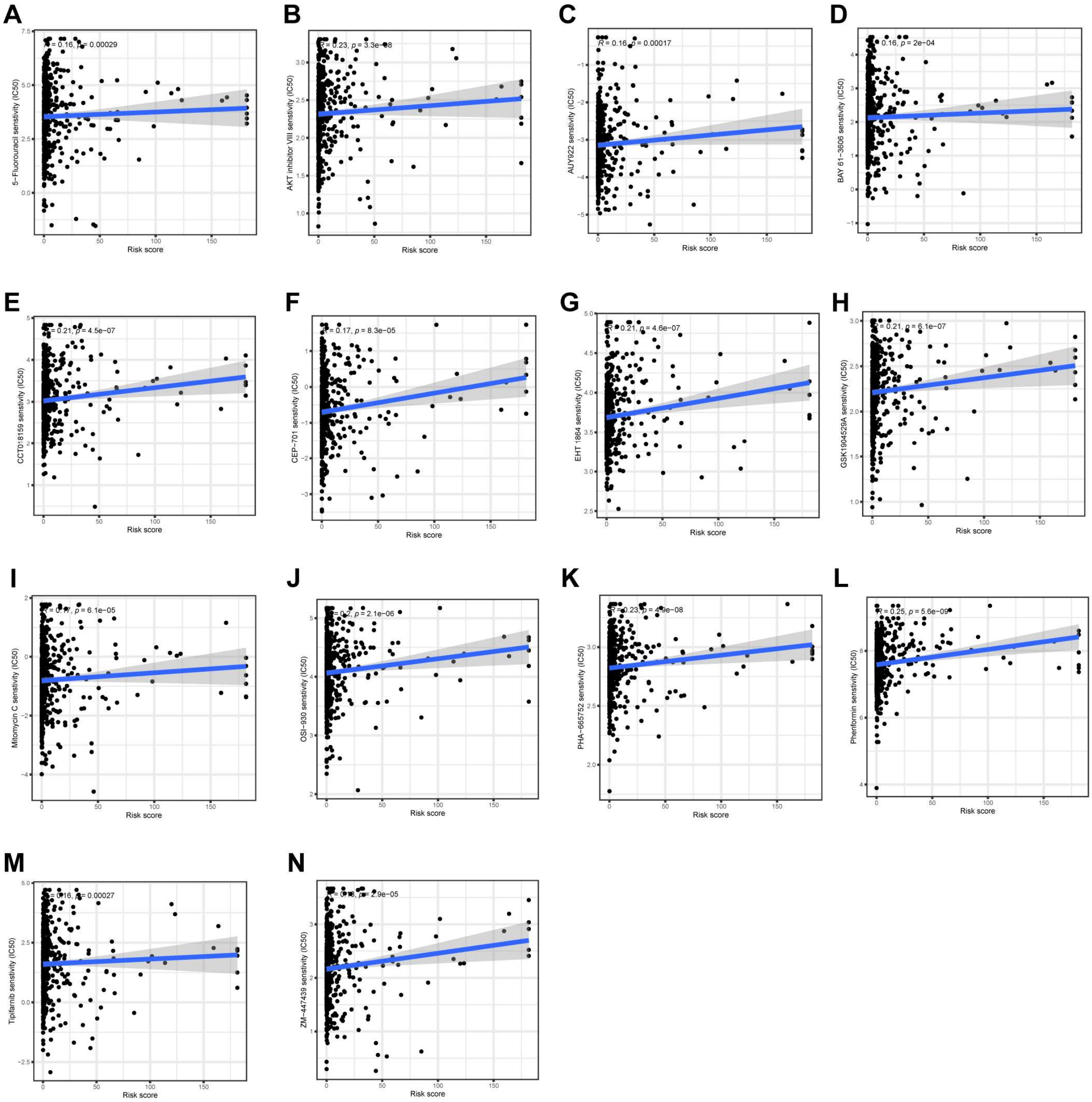

Supplementary Fig. S2 Drug susceptibility analysis. (A–N) The correlation between the IC50 values and risk scores of the fourteen aforementioned drugs
